# Mutagenesis of the Varicella-Zoster Virus Genome Demonstrates That VLT and VLT-ORF63 Proteins Are Dispensable for Lytic Infection

**DOI:** 10.3390/v13112289

**Published:** 2021-11-16

**Authors:** Shirley E. Braspenning, Robert Jan Lebbink, Daniel P. Depledge, Claudia M. E. Schapendonk, Laura A. Anderson, Georges M. G. M. Verjans, Tomohiko Sadaoka, Werner J. D. Ouwendijk

**Affiliations:** 1 Department of Viroscience, Erasmus MC, 3015 GD Rotterdam, The Netherlands; s.braspenning@erasmusmc.nl (S.E.B.); c.schapendonk@erasmusmc.nl (C.M.E.S.); l.anderson@erasmusmc.nl (L.A.A.); g.verjans@erasmusmc.nl (G.M.G.M.V.); 2 Department of Medical Microbiology, University Medical Center Utrecht, 3508 GA Utrecht, The Netherlands; R.J.Lebbink-2@umcutrecht.nl; 3 Institute of Virology, Hannover Medical School, 30625 Hannover, Germany; Depledge.Daniel@mh-hannover.de; 4 Division of Clinical Virology, Center for Infectious Diseases, Kobe University Graduate School of Medicine, Kobe 650-0017, Japan

**Keywords:** varicella-zoster virus, VLT, VLT-ORF63, CRISPR/Cas9, BAC mutagenesis

## Abstract

Primary varicella-zoster virus (VZV) infection leads to varicella and the establishment of lifelong latency in sensory ganglion neurons. Reactivation of latent VZV causes herpes zoster, which is frequently associated with chronic pain. Latent viral gene expression is restricted to the VZV latency-associated transcript (VLT) and VLT-ORF63 (VLT63) fusion transcripts. Since VLT and VLT63 encode proteins that are expressed during lytic infection, we investigated whether pVLT and pVLT-ORF63 are essential for VZV replication by performing VZV genome mutagenesis using CRISPR/Cas9 and BAC technologies. We first established that CRISPR/Cas9 can efficiently mutate VZV genomes in lytically VZV-infected cells through targeting non-essential genes ORF8 and ORF11 and subsequently show recovery of viable mutant viruses. By contrast, the VLT region was markedly resistant to CRISPR/Cas9 editing. Whereas most mutants expressed wild-type or N-terminally altered versions of pVLT and pVLT-ORF63, only a minority of the resulting mutant viruses lacked pVLT and pVLT-ORF63 coding potential. Growth curve analysis showed that pVLT/pVLT-ORF63 negative viruses were viable, but impaired in growth in epithelial cells. We confirmed this phenotype independently using BAC-derived pVLT/pVLT-ORF63 negative and repaired viruses. Collectively, these data demonstrate that pVLT and/or pVLT-ORF63 are dispensable for lytic VZV replication but promote efficient VZV infection in epithelial cells.

## 1. Introduction

Varicella-zoster virus (VZV) is a widespread human alphaherpesvirus and the causative agent of both varicella (chickenpox) and herpes zoster (HZ or shingles) [1]. During primary infection (varicella), VZV gains access to sensory ganglia where it establishes lifelong latency [2,3]. In one-third of infected individuals latent VZV will reactivate to cause HZ later in life, a condition often complicated by chronic pain (post-herpetic neuralgia) for which currently no effective treatment is available [4,5]. Although a potent subunit vaccine, Shingrix, has recently been licensed to prevent HZ in the elderly and immunocompromised [6], the latent virus itself is not targeted and thus reactivation events are not excluded. However, the development of therapeutics that eradicate the latent virus is hampered by a limited understanding of mechanisms governing latency and reactivation. 

During lytic infection VZV expresses at least 136 distinct polyadenylated RNAs, both coding and non-coding, that are expressed in a coordinated cascade [7,8]. By contrast, VZV latency is characterized by expression of the VZV latency-associated transcript (VLT) and, to a lesser extent, low-level expression of two fusion transcripts of VLT and RNA 63 (VLT63-1 and VLT63-2) [9,10]. VLT and VLT63s are also expressed as *True-Late* transcripts during lytic VZV infection. However, unlike the single VLT isoform and two VLT63 isoforms expressed during latency, numerous lytic VLT (_lyt_VLT) and VLT63 (_lyt_VLT63) isoforms are produced by the usage of alternative transcription start-sites, read-through transcription and alternative splicing. Whereas _lyt_VLT and _lyt_VLT63-2 encode VLT protein (pVLT), _lyt_VLT63-1 encodes for a pVLT-ORF63 fusion protein, in which canonical pORF63 is C-terminally linked to a N-terminal part of pVLT (118 of 136 aa) [10]. However, the function of pVLT and pVLT-ORF63 during lytic VZV infection remains elusive.

Similar to latency-associated transcripts of other alphaherpesviruses, the VLT locus is located partially antisense to ORF61 [the homologue of herpes simplex virus infected cell polypeptide 0 (*ICP0*)]. VLT RNA specifically downregulates the expression of RNA 61, which encodes the promiscuous transcriptional regulatory protein ORF61 (pORF61), in co-transfection experiments, suggesting that VLT may function in the establishment or maintenance of latency [9]. Ectopic in vitro expression of pVLT-ORF63 in latently VZV-infected human sensory neurons induces broad viral gene expression, suggesting that pVLT-ORF63 is a key regulator in the transition from latent to lytic infection [10]. However, the complexity of _lyt_VLT transcription and the genomic position of VLT antisense to the essential gene ORF61 [11,12], hinders complete deletion of the VLT locus from the viral genome and complicates studies into VLT function. 

Mutant herpesviruses can be generated using bacterial artificial chromosome (BAC)-based mutagenesis or by clustered regularly interspaced short palindromic repeats (CRISPR) CRISPR-associated protein 9 (Cas9) (CRISPR/Cas9) editing [13,14]. BAC-based mutagenesis remains the gold standard and allows for precise nucleotide insertions, deletions or substitutions to study viral protein functions, but are restricted to the genetic background of the parental BAC clone. By contrast, CRISPR/Cas9 editing results in the insertion or deletion of one to several nucleotides and can be used to edit viruses regardless of their origin, thereby providing a potential tool to combat herpesvirus infections in vivo. Furthermore, the application of CRISPR/Cas9 genome editing during lytic infection in vitro allows for direct competition between progeny viruses (natural selection), and hence the resulting viruses provide insight into the biological relevance of the targeted genomic region for viral replication. 

In this study, we investigated the role of pVLT and pVLT-ORF63 during lytic VZV infection in epithelial cells using mutant viruses obtained through complementary CRISPR/Cas9 genome editing and BAC mutagenesis. 

## 2. Materials and Methods

### 2.1. Cells and Viruses

Human retinal pigmented epithelial ARPE-19 cells were cultured in a 1:1 (*v*/*v*) mixture of DMEM (Lonza, Verviers, Belgium) and Ham’s F12 (Gibco, Breda, The Netherlands) supplemented with 10% heat-inactivated FCS, and 5% penicillin-streptomycin with L-glutamine (Lonza, Verviers, Belgium). Human melanoma MeWo cells (ATCC HTB-65) and MeWo-Cre cells expressing Cre-recombinase [15] were cultured in DMEM + GlutaMax-I (Thermo Fisher Scientific, Tokyo, Japan) supplemented with 8% FCS. VZV strain EMC-1 is a low-passage clinical isolate and served as the parental virus for CRISPR/Cas9-based gene editing. BAC-derived VZV was based on the pOka strain and generated as described below. Cell-free VZV was obtained from ARPE-19 cells, as described [16,17]. All cells and viruses were maintained in a humidified CO_2_ incubator at 37 °C. 

### 2.2. DNA Isolation from Varicella-Zoster Virus (VZV)-Infected Cells

VZV-infected ARPE-19 cells were subjected to DNA isolation using the QIAamp DNA mini kit (Qiagen, Venlo, The Netherlands) according to manufacturer’s instructions. DNA was eluted in 200 μL and used for PCR and Sanger sequencing.

### 2.3. RNA Isolation and cDNA Synthesis

VZV-infected cells were harvested in 1 mL Trizol (Thermo Fisher Scientific, Breda, The Netherlands), mixed with 200 μL chloroform and centrifuged for 15 min at 12,000× *g* at 4 °C. From the aqueous phase, RNA was isolated using the RNeasy Mini kit (Qiagen, Venlo, The Netherlands) according to manufacturer’s instructions, including on-column DNAse I treatment. RNA concentration was determined using a Nanodrop spectrophotometer, and residual DNA was removed using the Turbo DNA-free kit (Ambion, Breda, The Netherlands). For cDNA synthesis, maximum 5 µg RNA was reverse transcribed with Superscript IV reverse transcriptase (RT+) and oligo-dT primers (Thermo Fisher Scientific, Breda, The Netherlands). Negative control samples were obtained by performing the same cDNA synthesis reaction in the absence of reverse transcriptase (RT−).

### 2.4. Quantitative Polymerase Chain Reaction (PCR) Analysis

Taqman quantitative PCR (qPCR) was performed in duplicate on RT- and RT+ cDNA on a 7500 Taqman PCR system using 4× Taqman Fast Advanced Master mix (Applied Biosystems, Breda, The Netherlands). Primer-probe sets for VZV genes RNA 29, RNA 61, RNA 63, VLT2_3 and VLT3_4 have been described previously [9] and the primer-probe set for ORF60 was described and validated in this study. Primer sequences are given in Table S2. 

### 2.5. PCR and Sanger Sequencing

PCR was performed on extracted DNA or cDNA using AmpliTaq Gold DNA Polymerase (Thermo Fisher Scientific, Breda, The Netherlands) using primer pairs as indicated in Table S2 and the following PCR cycling parameters: initial denaturation at 95 °C for 10 min, followed by 40 cycles of 30 s at 95 °C, 30 s at 56 °C and 1 min at 72 °C. Extension was completed with a final step of 10 min at 72 °C. Resulting amplicons were either purified from gel using the Qiaquick Gel Extraction Kit (Qiagen, Venlo, The Netherlands) or used directly for sequence reaction, using the BigDye 3.1 Cycle Sequencing Kit (Applied Biosystems, Breda, The Netherlands) on the ABI PRISM 3130 XL Genetic Analyser.

### 2.6. Plasmid Construction and Generation of ARPE-19 Cells Stably Expressing pVLT

The pVLT coding sequence was amplified from cDNA of VZV EMC-1 infected ARPE-19 cells with NheI_VLT_Fw and VLT_Rv_BamHI primers (Table S2), cloned into the pcDNA3.1(+) backbone and the incorporated sequence was confirmed by Sanger sequencing. Stable cell lines ARPE-19 pVLT were then created by transfection with Genius transfection reagent (Westburg, Leusden, The Netherlands) of ARPE-19 cells at 70–80% confluency and subsequent selection with 1000 µg/mL geneticin (Gibco, Breda, The Netherlands) for at least 4 weeks. Expression of pVLT-encoding mRNA in the was confirmed by RT-qPCR as described above.

### 2.7. Generation of SpCas9-sgRNA Lentiviruses

crRNA target sequences (Table S1) were designed manually or using the CRISPOR website (http://crispor.tefor.net, accessed on 13 July 2017) and cloned as sgRNA downstream a human U6 promoter in the pSicoR-CRISPR-PuroR or pSicoR-CRISPR-BlastR vector [14]. All vectors were sequence verified by Sanger sequencing. For lentiviral transductions, viruses were produced in 293 T cells in 24-well plates using standard lentiviral production protocols and third-generation packaging vectors. 

### 2.8. Transduction and Selection of ARPE-19 Cells

ARPE-19 cells were seeded one day prior to lentiviral transduction in a 48-well plate at 70% confluency. The next day, lentiviral stocks were diluted 1:5 in culture medium containing 4 µg/mL polybrene and spin-inoculated (1000× *g*, 15 min) on the ARPE-19 cells. Two days after transduction, medium was replaced for selection medium containing either 0.94 µg/mL puromycin (InvivoGen, Toulouse, France) or 20 µg/mL blasticidin (InvivoGen, Toulouse, France) and stable cell lines were obtained by continuous selection for 3 weeks. To generate ARPE-19 cells expressing two sgRNAs, cells stably expressing the first sgRNA were lentivirally transduced to express the second sgRNA, followed by a second round of antibiotic selection.

### 2.9. Generation of CRISPR/Cas9-Based Mutant Viruses

ARPE-19 cells stably expressing one or two sgRNA(s) and SpCas9 were inoculated with cell-free VZV EMC-1 (MOI = 0.01) in a 12-well plate. Cell-free virus was harvested at 2 days post infection (dpi) and a 2-fold serial dilution was used to infect fresh monolayers of ARPE-19 cells in a 96-well plate. In case of sgRNA VLT-9, the obtained cell-free VZV was also serially diluted on ARPE-19 cells stably expressing pVLT. After 5–6 days incubation, plates were screened and wells containing a single plaque were passaged on fresh ARPE-19 cells in a 12-well plate. Obtained virus cultures were subjected to another 2 (double sgRNAs) or 3 (sgRNA VLT-9) rounds of plaque purification on ARPE-19 cells. The resulting viruses were analyzed by Sanger sequencing of the VLT target region. 

### 2.10. Illumina Sequencing of CRISPR/Cas9-Derived Mutant Viruses

ARPE-19 cells were infected with VZV EMC1-pVLTwt, EMC1-pVLTΔ1aa, EMC1-pVLTshift or EMC1-pVLTstop, and at 50–70% CPE infected cells were harvested. The cell pellet was resuspended in 200 µL PBS + 0.1% sodium deoxycholate, 50 µL 25 mM MgCl_2_ and 2.5 µL Omnicleave (Lucigen, Middleton, WI, USA) and incubated for 1 h at 37 °C and DNA was subsequently isolated by the QIAamp DNA mini kit (Qiagen, Venlo, The Netherlands) as described. Paired-end Illumina sequencing libraries were prepared from 100 ng DNA using the KAPA HyperPlus library preparation kit (Roche, Almere, The Netherlands) according to the manufacturer’s instructions with KAPA Unique Dual-Indexed Adapters Kit (Roche) for multiplexing. Libraries were pooled with unrelated samples and run on an Illumina MiSeq version 3 flowcell (2 × 300 cycles) (Illumina, San Diego, CA, USA). Resulting sequencing data were demultiplexed and trimmed to remove adapter sequences and low-quality 3’ nucleotides using TrimGalore (https://www.bioinformatics.babraham.ac.uk/projects/trim_galore/, v0.6.5). Trimmed sequence data were aligned against the VZV strain Dumas reference genome using bbmap (https://sourceforge.net/projects/bbmap/,) and parsed using SAMTools [18] and BEDTools [19]. Bam-readcount (https://github.com/genome/bam-readcount, v1.0.0), with the flag –d 1000000, was subsequently used to identify all reported SNPs and Indels prior to filtering using a custom script, variant_caller_v1.0.py, available at https://github.com/DepledgeLab/NAGATA.

### 2.11. Generation of Bacterial Artificial Chromosome (BAC) Mutant and Repaired Genomes by Redα/β-Mediated Linear Recombination

The pBpOka100798-104820 plasmid was generated by PCR amplification of the VLT locus (100,798–104,820; pOka [AB097933]) of pOka-BAC DNA [20] using primers pOka100798ecoF and pOka104820salR (Table S2) and subsequent cloning into pBlueScript II SK(-) (Agilent Technologies, Tokyo, Japan). The pBpOka100798-104820VLTM1I plasmid containing G->A mutation at position 102,824—changing the ATG (methionine; M) start codon of pVLT into ATA (isoleucine; I)—was generated from the pBpOka100798-104820 plasmid with primer VLTG3A (Table S2) using QuickChange Lightning Multi Site-Directed Mutagenesis Kit (Agilent Technologies) according to the manufacturer’s instruction. Next, a DNA fragment containing an ampicillin-resistant gene and *sacB* gene flanked by 102,501–102,550 and 104,448–104,500 of pOka was PCR amplified from the pST76A-SR plasmid [21] using primers pOka102501ampsacF and pOka104500ampsacR (Table S2) and subsequently transformed into GS1783 *E. coli* harboring pOka-BAC genome [22], resulting in the replacement of 102,551–104,447 of pOka-BAC by an ampicillin resistant gene and *sacB* gene (pOka-BACampsac1st). To generate the pOka-BACVLTM1I genome a DNA fragment of 100,798–104,820 of pOka amplified from pBpOka100798-104820VLTM1I plasmid was transformed into GS1783 harboring pOka-BACampsac1st, and followed by negative selection with 5% sucrose. The repaired BAC genome pOka-BACVLTM1IR was similarly generated based on the pOka-BACVLTM1I. Briefly, the 102,551–104,447 region of pOka-BACVLTM1I was replaced by an ampicillin-resistant gene and *sacB* gene in GS1783 *E. coli*, resulting in pOka-BACampsac2nd and the pOka-BACVLTM1Irev genome was then generated by transformation of a DNA fragment of 100,798–104,820 of pOka amplified from pBpOka100798-104820 plasmid into GS1783 pOka-BACampsac2nd, followed by negative selection with 5% sucrose. Finally, pOka-BACVLTM1I and pOka-BACVLTM1IR were purified using the Genopure Plasmid Maxi Kit (Roche Diagnostics, Tokyo, Japan), subjected to restriction fragment length polymorphism analysis using BamHI or EcoRI and Sanger sequencing of the recombined region.

### 2.12. Reconstitution of pOka-VLTM1I and pOka-VLTM1IR Viruses from BAC Genomes

VZV strain pOka-based VLTM1I and its repaired VLTM1IR recombinant viruses were reconstituted in MeWo cells by transfection of pOka-BACVLTM1I and pOka-BACVLTM1IR genomes using Lipofectamine2000 (Thermo Fisher Scientific) and the BAC cassette within the reconstituted viruses was subsequently removed in MeWo-Cre cells [23,24].

### 2.13. Immunofluorescence

ARPE-19 cells on glass coverslips were inoculated with freshly harvested cell-free VZV EMC-1 edited by sgRNA8-2 and incubated for 3 days at 37 °C. Infected cells were fixed with 4% paraformaldehyde (PFA), permeabilized for 10 min with 0.1% Triton X-100 in PBS, blocked with 5% goat serum diluted in PBS + 0.05% Tween and incubated with monoclonal mouse IgG1 anti-ORF8 antibody (1:100, generously provided by Dr. S. Jonjic, University of Rijeka, Rijeka, Croatia [16]) and monoclonal mouse IgG2b anti-VZV glycoprotein E antibody (MAB8612, Millipore, Amsterdam, The Netherlands) diluted PBS + 0.05% Tween 20 (PBS-T) overnight at 4 °C. Cells were washed with PBS-T and incubated with AF488-conjugated goat anti-mouse IgG2b and AF555-conjugated goat anti-mouse IgG1 antibodies (Thermo Fisher Scientific, Breda, The Netherlands) diluted 1:500 in PBS-T. Finally, the coverslips were washed, incubated for 5 min in PBS with Hoechst 33342 (1:1000, Life Technologies, 20 mM), washed in PBS and mounted using Prolong Gold Antifade Mounting medium (Thermo Fisher Scientific, Breda, The Netherlands). Stained cells were analyzed using a Zeiss LSM 700 confocal laser scanning microscope (Zeiss, Oberkochen, Germany) with a magnification of 100× or 400×. Photoshop CC 2021 software (Adobe, San Jose, CA, USA) was used to adjust brightness and contrast.

### 2.14. Infectious Focus Assay

ARPE-19 cells were plated in a 24-well plate and the next day cell monolayers were infected with 100 PFU/well of cell-free VZV. At 6 dpi, plates were washed and fixed with 4% PFA in PBS. Subsequently, cells were permeabilized with 0.1% Triton X-100 in PBS for 10 min, blocked with 5% normal goat serum in PBS-T for 30 min, incubated with mouse anti-VZV glycoprotein E antibody (MAB8612, Millipore, Amsterdam, The Netherlands) diluted 1:3200 in PBS-T overnight at 4 °C. The next day, the plates were washed with PBS-T, incubated for 1 h at room temperature with 1:200 biotinylated rabbit anti-mouse Ig antibody (DAKO, Amstelveen, The Netherlands) in PBS-T, washed and incubated for 1 h at room temperature with 1:300 streptavidin-HRP (DAKO, Amstelveen, The Netherlands) in PBS-T, washed once in PBS-T and twice with PBS. Finally, the signal was visualized using 3-amino-9-ethylcarbazole (AEC) as a substrate. Infectious foci were measured using the Immunospot S6 Ultimate UV Image Analyzer and foci size was determined using the Immunospot software (Cellular Technology Limited, Cleveland, OH, USA).

### 2.15. Cell-Associated VZV Titer

Monolayers of ARPE-19 cells in a 24-well plate were infected with 1000 PFU/well VZV, harvested at 3 dpi and titrated on ARPE-19 cells in 96-well plates using five-fold serial dilutions. After 3 days, infectious virus titers were calculated using the Spearman–Karber formula and expressed as TCID50 per cm^2^ cell culture.

### 2.16. Flow Cytometry

ARPE-19 cells were plated in a 96-well plate one day prior to infection. Cells were infected with 150 PFU/well cell-free virus and harvested at 24 h post infection (hpi), 48 hpi and 72 hpi. Subsequently, cells were fixed and permeabilized with BD Cytofix/Cytoperm, stained for VZV glycoprotein E (MAB8612, Millipore) in BD PermWash for 30 min at 4 °C, stained with secondary APC-conjugated goat anti-mouse Ig antibody (BD Biosciences, Vianen, The Netherlands) for 30 min at 4 °C and finally resuspended for analysis. Frequency of VZV-infected (APC-positive) cells was determined. Experiments were performed on a BD FACSLyric flow cytometer and the data was analyzed using FlowJo software (BD Biosciences).

### 2.17. Western Blotting

Mock- or VZV-infected ARPE-19 cells at 5 dpi were incubated in RIPA lysis buffer (0.01 M Tris-HCl [pH 7.4], 0.15 M NaCl, 1% sodium deoxycholate, 1% NP-40 and 0.1% SDS) on ice for 15 min, sonicated in a water bath for 10 min, and centrifuged at 20,000× *g* for 15 min. Proteins were separated on a 4–12% Nu-PAGE Gel (Thermo Fisher Scientific) and transferred onto PVDF membranes (0.2 µm). The membrane was blocked in 5% (*w*/*v*) skim milk in PBS + 0.1% Tween 20 at room temperature for 1 h and subsequently stained overnight at 4 °C with polyclonal rabbit anti-pVLT (1:2000) [9], polyclonal rabbit anti-pORF63 (1:30,000) [9] or monoclonal mouse anti-α-tubulin (1:30,000, clone B-5-1-2) antibodies diluted in blocking buffer. Next day, the membranes were stained for 1 h at room temperature with donkey anti-rabbit IgG HRP-linked or sheep anti-mouse IgG HRP-linked (GE Healthcare Bio-Sciences, Tokyo, Japan) antibodies diluted 1:3000 in blocking buffer. The signal was visualized using Chemi-Lumi One Super (Nacalai Tesque, Inc., Kyoto, Japan) and captured using LAS4000mini (GE Healthcare Bio-Sciences).

### 2.18. Statistical Analysis

Figures show individual data points or mean +/− SEM, all statistical analyses were performed with Graphpad Prism 9 software using the statistical test indicated in the figure legends, with * = *p* < 0.05, ** = *p* < 0.01, *** = *p* < 0.001. If no statistics are given, the difference was not significant.

## 3. Results

### 3.1. Successful Editing of Non-Essential VZV Genes by CRISPR/Cas9

**Figure 1:**
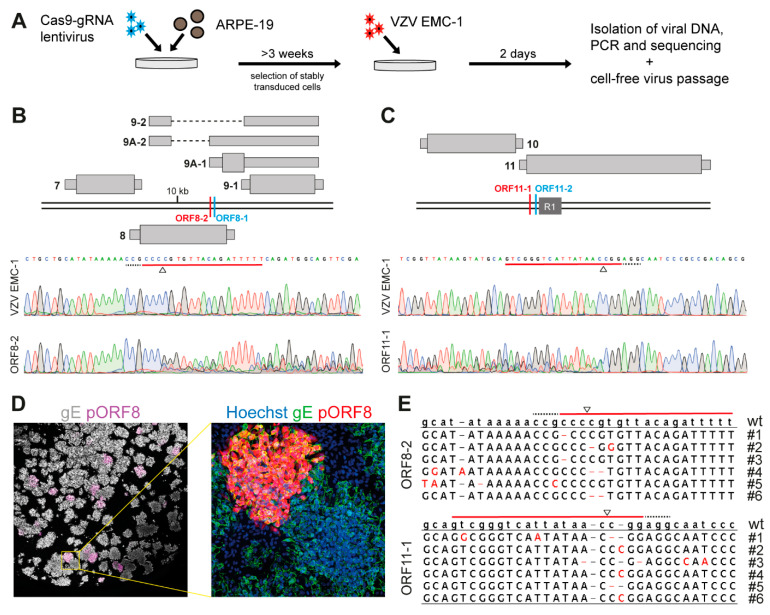
Editing of varicella-zoster virus (VZV) genomes using CRISPR/Cas9. (**A**) Experimental layout: ARPE-19 cells were transduced with a lentivirus encoding for both SpCas9 and a sgRNA, and selected for at least 3 weeks with appropriate antibiotics. Stably transduced cells were infected with cell-free VZV EMC-1 for two days, viral DNA was isolated and the target region was amplified by PCR and sequenced. (**B**,**C**) Top: Schematic illustration of the target region of ORF8 (**B**) and ORF11 (**C**): thick double black lines represent the VZV genome, currently annotated RNAs are depicted in grey—wide boxes are coding sequences (CDS), thin boxes are untranslated regions (UTRs) and dashed lines are intronic sequences—and DNA target sites of sgRNAs ORF8-1 and ORF8-2 (**B**) or sgRNAs ORF11-1 and ORF11-2 (**C**) are depicted. Bottom: Sanger sequencing traces showing the sgRNA target regions (red), PAM sequence (dashed black) and cleavage site with triangle for wild-type VZV EMC-1 DNA or from DNA extracted from VZV EMC-1 infected stably transduced ORF8-2 (**B**) and ORF11-1 cells (**C**). (**D**) Representative images of infected ARPE-19 cells with cell-free virus isolated from infected ORF8-2 cells stained by immunofluorescence for glycoprotein E (gray/green) and pORF8 (magenta/red). Nuclei were counterstained with Hoechst 33342 (blue), with 20× magnification (left) or 200× magnification (right). (**E**) Consensus Sanger sequences of 6 individual infectious foci after a single round of cell-free virus isolation and plaque purification following infection of stably transduced ORF8-2 (top) and ORF11-1 (bottom) cells. sgRNA target regions (red line), PAM sequence (dashed black line) and cleavage site with triangle are depicted.

To establish whether CRISPR/Cas9 can be used to edit VZV genomes during lytic infection, we initially targeted two VZV genes shown to be non-essential in cell culture: ORF8 and ORF11 [25,26]. To this end, we generated stable transduced human retina epithelial cells (ARPE-19) expressing both the *Streptococcus pyogenes* Cas9 (SpCas9) endonuclease and a single guide RNA (sgRNA) targeting the genomic region of interest (Figure 1A and Table S1). To counter the possibility of inefficient sgRNAs [27], we designed two sgRNAs for each ORF. We infected the stable cell lines with cell-free VZV (strain EMC-1), isolated DNA 2 days post-infection (dpi), and performed PCR and Sanger sequencing of the target regions (Figure 1A). Both ORF8 sgRNAs (ORF8-1 and ORF8-2), and one ORF11 sgRNA (ORF11-1) successfully induced editing of the VZV genome, as indicated by non-clonal Sanger traces around the sgRNA target site (Figure 1B,C). sgRNA ORF11-2 did not detectably induce VZV genome editing. To determine whether CRISPR/Cas9 edited VZV is viable and editing leads to functional disruption of viral protein expression, we isolated sgRNA ORF8-2 edited VZV, infected ARPE-19 cells and stained infectious foci for ORF8 protein (pORF8). While the majority of the foci completely lacked pORF8 expression, various levels of pORF8 were still detectable in some foci (Figure 1D). Sanger sequencing analysis on the mutant virus isolates (*n* = 6 per sgRNA), obtained after a single round of limited dilution and plaque purification, detected insertions or deletions (indels) in all analyzed isolates edited using sgRNA ORF8-2 or sgRNA ORF11-1 (Figure 1E). However, as CRISPR/Cas9 editing was applied during active lytic infection in the presence of a multitude of viral genomes, the edited populations after a single plaque purification still contained a mixture of genomes. 

**Figure 2:**
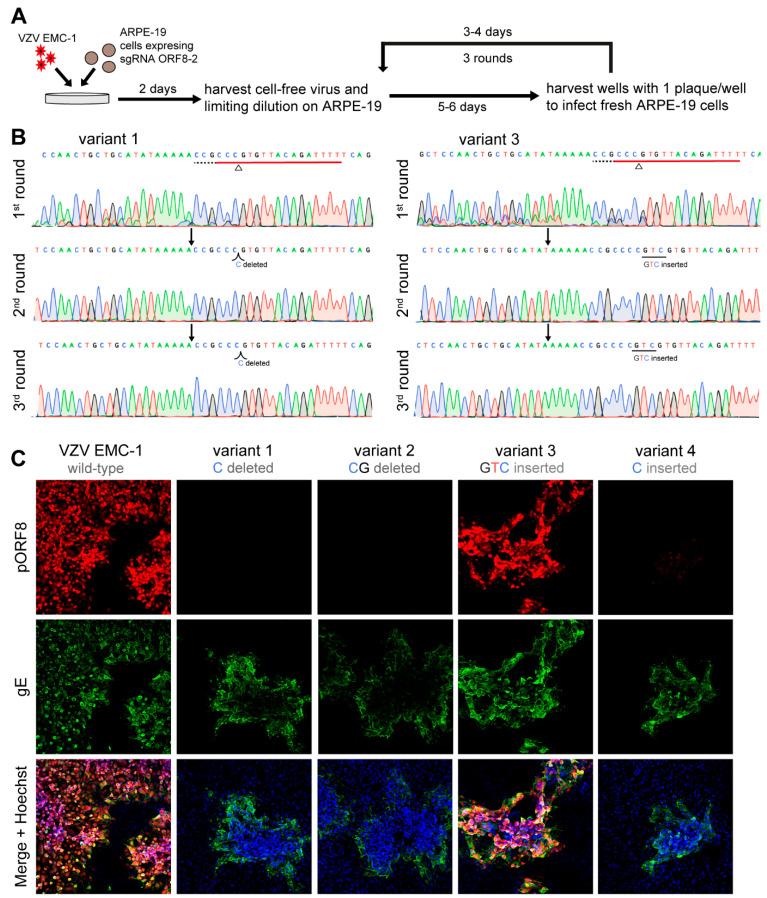
Plaque purification of VZV edited by gRNA ORF8-2. (**A**) Experimental design: Stably transduced ARPE-19 cells expressing sgRNA ORF8-2 were infected with VZV EMC-1. Two days post-infection, cell-free virus was harvested, and subjected to three subsequent rounds of plaque purification. (**B**) Sanger sequencing after each round of plaque purification for variant 1 and 3 showing the sgRNA target regions (red), PAM sequence (dashed black), cleavage site with triangle and identified mutation. (**C**) Representative images of ARPE-19 cells infected with VZV EMC-1 or 4 ORF8 variant viruses derived from sgRNA ORF8-2 cells by plaque purifications stained by immunofluorescence for glycoprotein E (green) and pORF8 (red). Nuclei were counterstained with Hoechst 33342 (blue), with 10× magnification.

To establish a workflow for the isolation of near-clonal mutant virus populations using CRISPR/Cas9 editing, we infected gRNA ORF8-2 expressing cells with VZV EMC-1, isolated the cell-free virus and performed three successive rounds of cell-free virus isolation, limiting dilution and plaque purification (Figure 2A). Sanger sequencing at each intermediate population showed that after 2–3 rounds of plaque purification, sequence traces were clonal, suggesting highly pure (or clonal) virus population (Figure 2B). In total, we recovered four mutant VZV isolates, of which three contained disruption of pORF8, as predicted by their nucleotide sequences and confirmed by immunofluorescence staining of ARPE-19 cells infected by these viruses. (Figure 2C). Thus, we established a CRISPR/Cas9 genome editing approach that can be applied to target VZV genomes during lytic infection and showed that mutant viruses can be purified by successive plaque purifications.

### 3.2. The VZV Latency-Associated Transcript (VLT) Locus Is Resilient to CRISPR/Cas9 Genome Editing

**Figure 3:**
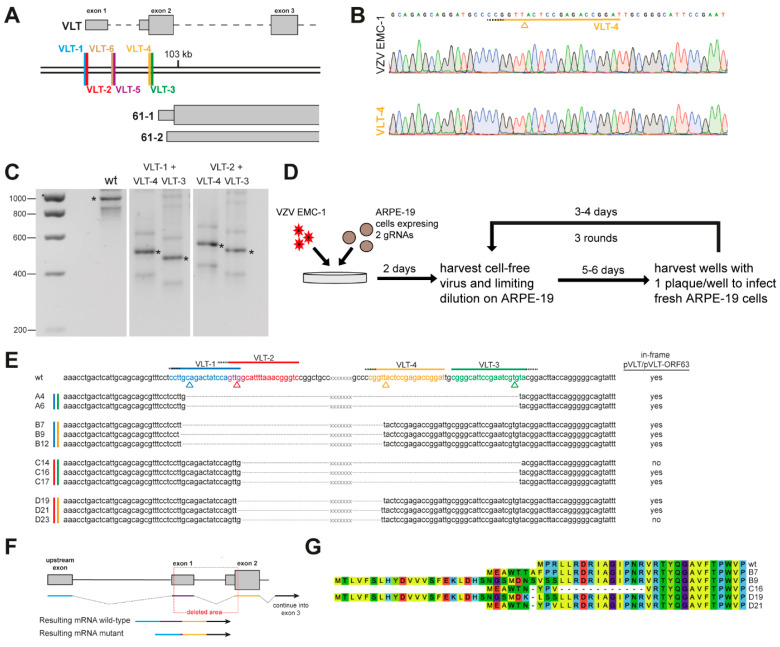
Generation of VZV latency-associated transcript (VLT) mutant viruses using combinations of two different sgRNAs. (**A**) Schematic illustration of the VLT target region: thick double black lines represent the VZV genome, annotated RNAs are depicted in grey—wide boxes are coding sequences (CDS), thin boxes are untranslated regions (UTRs) and dashed lines are intronic sequences—and the DNA target sites of sgRNAs VLT-1 to VLT-6 are depicted in differentially colored lines. (**B**) Sanger sequencing traces showing the sgRNA target region (yellow), PAM sequence (dashed black) and cleavage site (colored triangle) for wild-type VZV EMC-1 DNA (top) and DNA extracted from VZV EMC-1 infected stably transduced VLT-4 cells (bottom). (**C**) PCR products from VLT upstream exon C to VLT exon 2 on DNA isolated from parental ARPE-19 cells or from cells stably transduced with a combination of sgRNAs as indicated 2 days post-infection with VZV EMC-1. Correct band is indicated by an asterisk, additional bands result from duplication of primer binding site in R5. (**D**) Experimental design: Stably transduced ARPE-19 cells expressing two sgRNAs targeting the VLT region were infected with VZV EMC-1. Two days post-infection, cell-free virus was harvested, and subjected to three subsequent rounds of plaque purification. (**E**) VZV DNA sequence around sgRNA target sites of isolated plaque purified viruses divided into 4 groups: A—combination of sgRNA VLT-1 and sgRNA VLT-3, B—combination of sgRNA VLT-1 and sgRNA VLT-4, C—combination of sgRNA VLT-2 and VLT-3, D—combination of sgRNA VLT-2 and VLT-4. Dashed lines represent deleted nucleotides, whereas ‘xxx’ signifies the region in between the sgRNAs target sequences deleted in all mutants. PAM sequences (dashed black) and expected cleavage sites (colored triangles) are indicated. (**F**) Schematic illustration of VLT transcript generation from wild-type or mutant viruses from any upstream VLT exon (blue line), into exon 1 (purple line), exon 2 (orange line) and the remainder of VLT (black arrow). (**G**) Alignment of proteins generated in mutant viruses by in-frame splicing of an upstream ATG with the remainder of pVLT/pVLT-ORF63. Amino acids are colored to aid visual comparison.

To target the VZV VLT locus and generate VLT mutant viruses, we designed six sgRNAs targeting three distinct regions of VLT that are present in all _lyt_VLT isoforms, as well as in _lyt_VLT63-1 and _lyt_VLT63-2 (Figure 3A and Table S1). Two sgRNAs—VLT-1 and VLT-2—targeted VLT exon 1 and potentially disrupt splicing from upstream exons into VLT exon 1. Two sgRNAs—VLT-3 and VLT-4—were directed close to the only in-frame pVLT ATG start codon in located VLT exon 2 to interfere with pVLT/pVLT-ORF63 expression. Finally, two sgRNAs—VLT-5 and VLT-6—targeted the first intron of VLT, into which a large GFP-expression cassette was inserted previously by BAC-based mutagenesis [28], suggesting mutations in this region are tolerated. We generated stable cells lines for each sgRNA, infected these cells with cell-free VZV EMC-1 and performed Sanger sequencing on viral DNA extracted at 2 dpi. Strikingly, none of the sgRNAs induced detectable VZV genome editing (Figure 3B), independent of the multiplicity of infection or viral DNA replication (data not shown). 

Conformational structures of the genome might render mammalian genes less accessible to endonucleases and thus difficult to target by CRISPR/Cas9 [29,30]. For some of those genes, combination of two low-efficient sgRNAs leads to efficient editing of the target region [31,32]. Hence, we generated four cell lines stably expressing a combination of two sgRNAs; one in exon 1, and the other in exon 2 (Figure 3A,C). VZV EMC-1 infection of all double sgRNA expressing cell lines lead to efficient deletion of VLT exon 1, intron 1 and part of exon 2 (±450 bp) from the VZV genome, as indicated by a smaller PCR product of the extracted viral DNA (Figure 3C). Next, we infected these double sgRNA cell lines to generate four groups of VLT mutant viruses (hereafter named A–D), followed by three successive rounds of cell-free virus isolation, limiting dilution and plaque purifications on parental ARPE-19 cells (Figure 3D). We obtained 12 virus isolates—three for each combination—on which we performed Sanger sequencing to determine the DNA sequence of the VLT region. Eleven out of 12 isolates contained deletions within the VLT region that correspond to the combination of sgRNAs used; within the four groups, 1–2 nucleotide differences were observed between mutant viruses (Figure 3E). In silico predictions on sequenced _lyt_VLT/_lyt_VLT63 transcripts showed that in 9 out of 11 isolates an upstream ATG start codon was positioned in-frame with the remainder of canonical pVLT/pVLT-ORF63 coding sequences, potentially resulting in N-terminally extended and altered proteins (Figure 3F,G). Importantly, two mutant isolates—C14 and D23—expressed _lyt_VLT/_lyt_VLT63 transcripts that were not predicted to encode for pVLT/pVLT-ORF63 (or variants thereof) (Figure 3E). 

### 3.3. Growth Characterization of VZV VLT Mutant Viruses

**Figure 4:**
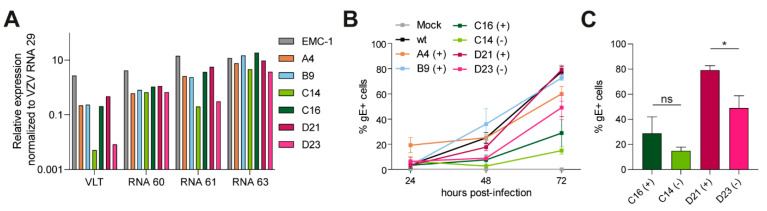
Characterization of VZV VLT mutant viruses generated using combinations of two sgRNAs. (**A**) Relative expression level of VLT RNAs, RNA 60, RNA 61 and RNA 63 in ARPE-19 cells asynchronously infected with either wild-type VZV EMC-1 or mutants A4, B9, C14, C16, D21 and D23. Expression levels were normalized to VZV RNA 29 to compensate for differences in infection. (**B**) Representative growth curves of wild-type virus, one mutant per group and two pVLT/pVLT-ORF63(-) viruses by flow cytometry of VZV infected cells (*n* = 2 independent experiments, *n* = 3 technical replicates). Percentages shown are VZV glycoprotein E (gE)—positive cells at 24, 48 and 72 hpi. None of the mutant viruses significantly differed from wild-type (one-way ANOVA). (**C**) Comparison between two pVLT/pVLT-ORF63(+) viruses (C16 and D21) and their pVLT/pVLT-ORF63(-) counterparts (C14 and D23) at 72 hpi. ns: not significant and * *p* < 0.05 by unpaired Student’s *t*-test.

Next, we analyzed the expression levels of _lyt_VLT and neighboring VZV transcripts in VZV VLT mutant virus isolates compared to VZV EMC-1 by RT-qPCR. Relative expression levels of _lyt_VLT/_lyt_VLT63 RNAs were reduced for all mutants compared to parental VZV EMC-1, especially in pVLT/pVLT-ORF63(-) isolates, C14 and D23 (Figure 4A). However, expression of RNA 60 and RNA 61 was also reduced in all mutant viruses, whereas expression of RNA 63 was not affected (Figure 4A). Together, these data show that large deletions in the VLT region impact relative expression of VLT and also its flanking genes, presumably by affecting regulatory sequences in the 5’UTR of RNA 60 and/or 3’UTR of RNA 61. 

Subsequently, we compared viral growth of these VLT mutant isolates and VZV EMC-1 in ARPE-19 cells by flow cytometry. We observed substantial variability between biological replicates, which was independent of the virus used and most likely caused by the high particle to PFU ratio inherent to cell-free VZV stocks [33,34]. Although none of the mutant virus isolates significantly differed from VZV-EMC-1 (Figure 4B), the two pVLT/pVLT-ORF63(-) mutants (C14 and D23) appeared to be impaired in replication compared to pVLT/pVLT-ORF63(+) counterparts from the same group (C16 and D21 respectively) (Figure 4C). However, since these mutant viruses contain relatively large deletions in the VLT region that also affect expression of VLT, RNA 60, and RNA 61, we cannot exclude the possibility that the observed phenotype is partially unrelated to the absence of pVLT/pVLT-ORF63. 

### 3.4. Generation of VZV pVLT/pVLT-ORF63 with Indel Mutations

**Figure 5:**
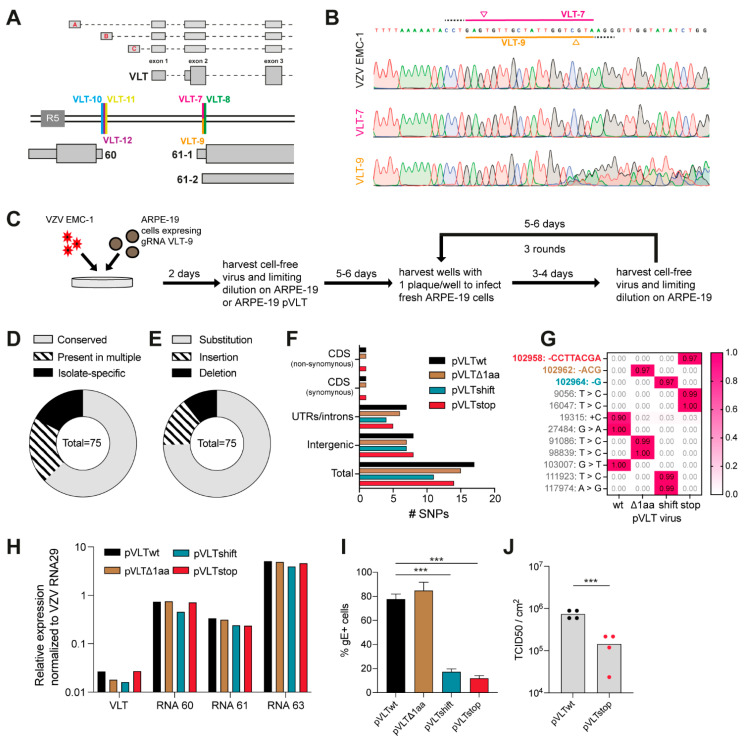
Generation of VZV pVLT/pVLT-ORF63 mutant viruses using a single sgRNA. (**A**) Schematic illustration showing the sgRNA target sites in the VLT region: thick double black lines represent the genome, annotated RNAs are depicted in grey—wide boxes are coding sequences (CDS), thin boxes are untranslated regions (UTRs) and dashed lines are intronic sequences—and the DNA target sites of sgRNAs VLT-7 to VLT-12 are depicted in differentially colored lines. Upper three transcripts represent most abundant alternative upstream exons used during lytic infection—named A-B-C and described in [9]. (**B**) Sanger sequence traces showing the sgRNA VLT-7/VLT-9 target region with PAM sequence (dashed black) and cleavage site (colored triangle) for wild-type VZV EMC-1 DNA (top) or DNA extracted from VZV EMC-1 infected stably transduced VLT-7 cells (middle) and VLT-9 cells (bottom). (**C**) Experimental layout: Stably transduced ARPE-19 cells expressing sgRNA VLT-9 were infected with VZV EMC-1. Two days post-infection, cell-free virus was harvested, and subjected to plaque purification on either ARPE-19 or ARPE-19 pVLT cells. Recovered viruses when then purified by three subsequent rounds of plaque purification. (**D**–**G**) Illumina sequencing of viral DNA of four selected mutant virus isolates identified 75 SNPs compared to reference. (**D**) Distribution of SNPs conserved between all four isolates (*n* = 47), SNPs present in multiple isolates (*n* = 15) and isolate-specific SNPs (*n* = 13). (**E**) Type of SNPs identified in all isolates, substitutions (*n* = 56), insertions (*n* = 11) or deletions (*n* = 8). (**F**) Location of SNPs and number of SNPs outside the VLT coding sequence for each mutant viral isolate. (**G**) Proportion of reads of isolate-specific mutations for each mutant viral isolates, with VLT mutations highlighted in bold and colored: pVLTΔ1aa—brown, pVLTshift—blue and pVLTstop—red. (**H**) Relative expression level of VLT RNAs, RNA 60, RNA 61 and RNA 63 in ARPE-19 cells asynchronously infected with either pVLTwt, pVLTΔ1aa, pVLTshift or pVLTstop. Expression levels were normalized to VZV RNA 29 to compensate for differences in infection. (**I**) Percentage VZV glycoprotein E (gE)–positive cells by flow cytometry for pVLTwt, pVLTΔ1aa, pVLTshift or pVLTstop at 72 hpi. Representative data for *n* = 2 independent experiments, *n* = 3 technical replicates. *** *p* < 0.001 by unpaired Student’s *t*-test. (**J**) Cell-associated VZV titers of monolayers infected with wt (black dot) or stop (red dot) virus at 72 hpi as measured by TCID50 per cm^2^. *** *p* < 0.001 by unpaired Student’s *t*-test.

To generate indel mutations within VLT, rather than larger deletions, we designed two additional sets of sgRNAs directed to the VLT locus. One set (*n* = 3 sgRNAs, designated VLT-7, -8 and -9) targets the 3’end of VLT exon 2, and could alter the pVLT/pVLT-ORF63 protein sequence (Figure 5A and Table S1). The other set (*n* = 3 sgRNAs, designated VLT-10, -11 and -12) targets one of the upstream exons of _lyt_VLT/_lyt_VLT63 [7]; presumably, deletion of only one of multiple possible alternative upstream exons would be compatible with VZV viability, as many other _lyt_VLT/_lyt_VLT63 variants remain available (Figure 5A and Table S1). Stable cell lines expressing these single sgRNAs and SpCas9 were infected with cell-free VZV EMC-1 and the viral DNA was sequenced at 2 dpi. None of the sgRNAs targeting the upstream _lyt_VLT/_lyt_VLT63 exon B induced any detectable mutations, nor did 2 out of 3 sgRNAs targeting VLT exon 2. Only a single sgRNA targeting exon 2 (VLT-9), induced efficient CRISPR/Cas9-based VZV genome editing (Figure 5B). 

Given that our pVLT/pVLT-ORF63(-) viruses with genomic deletions were impaired in replication compared to the corresponding pVLT/pVLT-ORF63(+) viruses (Figure 4), we performed an additional first round of limiting dilution of the cell-free virus obtained from VZV infection of the sgRNA VLT-9 cell line on either parental ARPE-19 cells or on ARPE-19 cells stably expressing pVLT (ARPE-19 pVLT), to complement for the potential loss of pVLT in trans (Figure 5C). Interestingly, all virus isolates recovered from parental ARPE-19 cells contained either no mutations in the VLT region or lacked three nucleotides, leading to an in-frame deletion of 1 amino acid (threonine 35). By contrast, virus isolates recovered from ARPE-19 pVLT cells contained other pVLT/pVLT-ORF63 mutations, such as frame-shift mutations or the formation of a premature stop codon, resulting in the loss of pVLT/pVLT-ORF63 coding potential (Table 1). We selected four viruses for further experiments and named them after their mutation; isolate A-VLT-5—pVLTΔ1aa, isolate P-VLT-5—pVLTshift, isolate P-VLT-8—pVLTstop, and the co-purified isolate A-VLT-7 with wild type VLT sequence—pVLTwt. 

Short-read Illumina sequencing of VZV pVLTwt, pVLTΔ1aa, pVLTshift and pVLTstop genomes identified 75 SNPs (Table S3) compared to the reference VZV Dumas DNA sequence (Genbank: NC_001348.1). The majority of these SNPs (*n* = 47) were conserved between all four VZV isolates and thus likely represent SNPs already present in the parental EMC-1 virus (Figure 5D). Another 15 SNPs were present in more than one VZV isolate but not shared between all, whereas 13 SNPs were specific to a single VZV isolate (Figure 5D). Most SNPs were substitutions, with insertions or deletions accounting for approximately one quarter of all SNPs (Figure 5E). The majority were located in intergenic regions, UTRs or introns, while only 23 non-VLT related SNPs affected the CDS of another VZV ORF (Figure 5F and Table S3). Most of these SNPs result in synonymous mutations and do not, therefore, impact the viral protein coding potential. One nonsynonymous SNP in the pVLTwt isolate resulted in a frameshift replacing the C-terminal 7AA with 11AA in pORF13, whereas pORF11 is extended by 3AA in the pVLTstop isolate (Table S3). Importantly, the high proportion of reads harboring the expected mutation for each VZV isolate in VLT exon 2 (Figure 5G), in the absence of any remaining contaminating wild-type VLT sequences in VZV pVLTΔ1aa, pVLTshift and pVLTstop, indicates clonality in this region. In summary, these Illumina DNA-sequencing data show CRISPR/Cas9 edited viruses can be efficiently isolated by repeated plaque purifications, yielding highly pure mutant viruses. 

In contrast to the viruses with large deletions in the VLT locus (Figure 4A), expression of VLT RNAs, RNA 60, RNA 61 and RNA 63 was not affected in VZV pVLTΔ1aa, pVLTshift and pVLTstop viruses compared to VZV pVLTwt (Figure 5H). Concordant with the data obtained for the viruses with large deletions, growth data for pVLT/pVLT-ORF63(+) viruses (pVLTwt and pVLTΔ1aa) and pVLT/pVLT-ORF63(−) viruses (pVLTshift and pVLTstop), showed that pVLT/pVLT-ORF63(−) viruses with indel mutations were significantly impaired in replication compared to pVLT/pVLT-ORF63(+) viruses at 72 hpi (Figure 5I). Furthermore, titration of cells infected with either the pVLTwt or pVLTstop virus at 72 hpi showed a significant reduction in virus titer for the pVLT/pVLT-ORF63(−) virus (Figure 5J). Thus, the loss of pVLT/pVLT-ORF63 coding potential impairs lytic VZV infection in ARPE-19 cells.

### 3.5. Generation of VZV pVLT/pVLT-ORF63 Mutant Viruses Using BAC Mutagenesis

**Figure 6:**
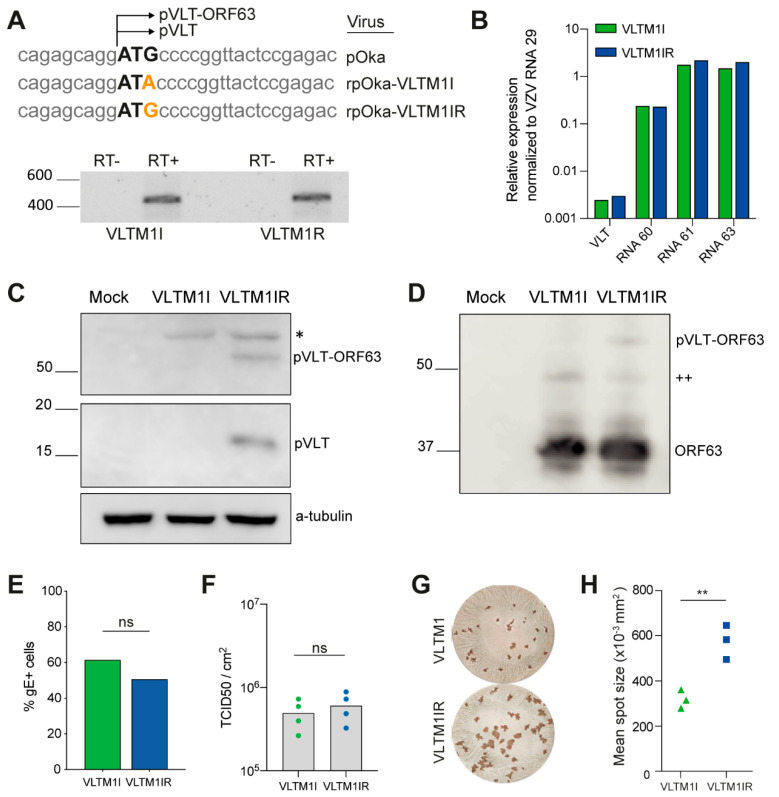
Generation of VZV pVLT/pVLT-ORF63 mutant viruses using BAC mutagenesis. (**A**) Top: Nucleotide sequence of VZV pOka, rpOka-VLTM1I and rpOka-VLTM1IR of the pVLT/pVLT-ORF63 ATG start codon in black, mutations in orange and its flanking regions and bottom: RT-PCR on RNA isolated from ARPE-19 cells infected with VZV VLTM1I or VLTM1IR using primers located in VLT exon 1 and exon 5. RT-/RT+: reverse transcriptase omitted or added in cDNA synthesis. (**B**) Relative expression level of VLT RNAs, RNA 60, RNA 61 and RNA 63 in ARPE-19 cells asynchronously infected with VLTM1I or VLTM1IR. Expression levels were normalized to VZV RNA29 to compensate for differences in infection. (**C**,**D**) Western blots on lysates of mock-, VZV VLTM1I- or VZV VLTM1IR-infected ARPE-19 cells stained with anti-pVLT, anti-alpha-tubulin (**C**) and anti-pORF63 (**D**) antibodies. Bands for pVLT and pVLT-ORF63 are indicated, * signifies an nonspecific band detected in all virus-infected cells, ++ indicates pORF63-N+ [10]. (**E**) Percentage VZV glycoprotein E (gE)–positive cells by flow cytometry of VZV VLTM1I and VLTM1IR in ARPE-19 cells at 72 hpi. Representative data for *n* = 2 independent experiments, *n* = 3 technical replicates. ns, not significant by unpaired Student’s *t*-test. (**F**) Cell-associated VZV titers of monolayers infected with VLTM1I or VLTM1IR virus at 72 hpi as measured by TCID50 per cm2, ns: not significant by unpaired Student’s *t*-test. (**G**,**H**) Infectious focus assay of VZV VLTM1I and VLTM1IR-infected ARPE-19 cells at 6 days post-infection (*n* = 2 independent experiments, 4 replicates each). (**G**) Representative images of infected wells stained for VZV gE by immunohistochemistry and (**H**) Mean spot size (×103 mm^2^) of VLTM1I (green) and VLTM1IR (blue) viruses per well, ** *p* < 0.01 by unpaired Student’s *t*-test.

The currently available anti-pVLT antibody recognizes the first 20 amino acids of pVLT [9], thereby prohibiting formal confirmation of the absence of pVLT/pVLT-ORF63 in the aforementioned VLT mutant viruses: the large deletions in the first set of VLT mutants (Figure 3) disturb the antibody binding site but potentially encode a N-terminal altered variant of pVLT/pVLT-ORF63, whereas in the set with VLT mutants generated by sgRNA VLT-9 (Table 1 and Figure 5) the stop codon is located 14 amino acids downstream of the antibody binding site. BAC-based mutagenesis is widely used to generate mutant herpesvirus genomes, and its power resides in studying viral protein functions at nucleotide-level precision. Thus, to create a mutant virus in which the absence of pVLT/pVLT-ORF63 could be confirmed and to substantiate our findings, we performed complementary BAC-based mutagenesis. We constructed a recombinant virus from the parental Oka genome cloned into a bacterial artificial chromosome (pOka-BAC) [20] in which the pVLT/pVLT-ORF63 start codon (ATG) is replaced for an isoleucine encoding codon (ATA), and its corresponding repaired virus, hereafter named VLTM1I and VLTM1IR, respectively (Figure 6A). We confirmed expression of VLT RNA in ARPE-19 cells infected with VZV VLTM1I or VLTM1IR by RT-PCR and subsequent Sanger sequencing (Figure 6A) and showed by cDNA qPCR that relative expression levels of VLT RNAs, RNA 60, RNA 61 and RNA 63 were similar between VLTM1I and VLTM1IR viruses (Figure 6B). Western blotting confirmed the absence of both pVLT and pVLT-ORF63 in VZV VLTM1I-infected cells (Figure 6C), whereas VLTM1I and VLTM1IR expressed pORF63 at comparable levels (Figure 6D). Unlike the CRISPR/Cas9-based pVLT/pVLT-ORF63 mutant viruses, infection of ARPE-19 cells with VLTM1I and VLTM1IR resulted in comparable frequencies of infected cells and cell-associated virus titers (Figure 6E,F). However, VLTM1I produced significantly smaller infectious foci compared to VLTM1IR at 6 dpi (Figure 6G,H). Possibly, the genetic background of CRISPR/Cas9-derived (EMC-1) and BAC-derived (pOka) viruses influences the rate of cell-to-cell spread, syncytia formation or induction of cell death. Indeed, we observed a marked difference in infectious focus morphology between CRISPR/Cas9-derived (EMC-1 based) and BAC-derived (pOka based) viruses (Figure S1). Together these data show that while pVLT and pVLT-ORF63 are dispensable for lytic VZV replication in ARPE-19 cells, their absence may reduce the efficiency of VZV spread in epithelial cells.

## 4. Discussion

VZV latency in sensory neurons is defined by selective expression of VLT and VLT-ORF63 fusion transcripts [9,10]. VLT and VLT-ORF63 RNAs are unique as they are the only gene products that are present throughout the entire viral life cycle—during both lytic and latent phases. VLT and VLT63-1 encode for proteins that are expressed during lytic infection: pVLT, a protein of unknown function, and pVLT-ORF63, likely a key regulator in the transition from latency to lytic infection [10]. Here, we studied the role of pVLT and/or pVLT-ORF63 during lytic VZV infection in epithelial cells using complementary CRISPR/Cas9- and BAC-based mutagenesis. We demonstrated that CRISPR/Cas9 editing of non-essential VZV genes leads to functional protein disruption, but that editing of the VLT region during lytic VZV infection is inefficient and favors the selection of pVLT/pVLT-ORF63 coding virus mutants. Additionally, VZV mutants lacking pVLT and pVLT-ORF63 coding potential, generated by either CRISPR/Cas9- or BAC-based mutagenesis, were mildly impaired in virus replication in ARPE-19 cells. Collectively, these data indicate that pVLT and/or pVLT-ORF63 are dispensable for lytic VZV replication, but the presence of these viral proteins promotes efficient VZV infection in ARPE-19 epithelial cells.

Our study reports on locus-specific VZV genome editing using CRISPR/Cas9 technology and subsequent purification of mutant virus isolates. Non-essential VZV genes ORF8 and ORF11 could be efficiently targeted in lytically VZV-infected cells and produced infectious mutant progeny virus. The VLT locus, however, proved to be difficult to target, and no genome editing was observed when using 11 of 12 single sgRNAs targeting the VLT locus. It is unclear what causes this resistance of the VLT region to CRISPR/Cas9 genome editing, as targeting essential herpesvirus genes is possible and typically results in the outgrowth of escape mutants that carry in-frame deletions [14]. Many cellular factors could influence the binding of the SpCas9-sgRNA complex to mammalian genomes, such as chromatin accessibility, nucleosome positioning and certain chromatin modifications [13,35,36]. Although herpesviral genomes are immediately chromatinized upon nuclear entry, viral proteins actively reduce histone occupancy leading to an open chromatin structure of the viral genome during lytic infection. This, combined with the efficient editing of the VLT locus using two sgRNAs, suggests that impaired chromatin accessibility or binding of the SpCas9-sgRNA complex to the viral genome does not contribute to the relative resistance of the genomic VLT region. Alternatively, the VLT region may contain unknown essential regulatory elements that require perfect repair by the cellular DNA damage repair machinery in order to produce infectious progeny. 

The CRISPR/Cas9 system provides the opportunity to edit clinical isolates [37], enabling studies of gene function in different genetic backgrounds, and to perform in vitro mutagenesis of viral genomes. Recovered genomic sequences are driven by selection (i.e., pressure during replication in permissive cell types) and, therefore, provide valuable insights into the importance of specific loci or favored mutation types. Most recovered virus isolates generated with the double sgRNA approach contained an upstream ATG start codon in-frame with the remainder of the pVLT/pVLT-ORF63 open reading frame, which suggests that the presence of N-terminally altered versions of these proteins is favored over their absence. Similarly, using CRISPR/Cas9 editing with sgRNA VLT-9, we were only able to obtain pVLT/pVLT-ORF63(-) viruses when rescued on cells constitutively expressing pVLT, suggesting that the presence of pVLT in trans provides an important survival advantage to mutant viruses. Indeed, we observed that pVLT/pVLT-ORF63(-) VZV was viable, but attenuated in growth compared to pVLT/pVLT-ORF63(+) VZV. 

Cell cultures are oversimplified representations of the host environment, and thus requirements for survival in vivo can substantially differ from those in vitro. Indeed, the phenotype of single-ORF deletion VZV mutants can be markedly different between cell culture and skin tissue culture or xenograft models [38,39,40,41,42], as illustrated by skin-specific growth defects of ORF7-deletion viruses and combined T-cell- and skin-specific growth defects of ORF67-deletion viruses [11,43]. Therefore, future studies in these skin explant and xenograft models are of interest to determine the role of pVLT/pVLT-ORF63 in organ-specific lytic VZV replication in vivo. Similarly, the recent development of human neuron models to study VZV latency and reactivation in vitro [10,44,45] enables future studies to determine the role of pVLT and pVLT-ORF63 in the establishment, maintenance and egress from latency. The latter is of particular interest, because we have recently shown that ectopic expression of VLT63-1—encoding pVLT-ORF63—induces broad viral gene expression from latent VZV genomes [10]. 

Currently available antivirals and vaccines that counteract and prevent VZV disease, leave the latent VZV burden intact. CRISPR/Cas9, besides being a versatile tool for research, also holds promising therapeutic potential for the treatment of persistent virus infections, as illustrated by the application to excise the integrated SIV genome in vivo [46,47]. Additionally, several studies showed successful targeting of herpesvirus genomes with either CRISPR/Cas9 or other programmable endonucleases, such as reducing latent EBV [14,48], latent KSHV [49], quiescent and latent HSV-1 genomes in vitro and in vivo [13,50] and, importantly, reducing herpes keratitis occurrence in vivo [23]. In this study, we showed that in vitro editing of VZV genomes during lytic infection is feasible. Although the VLT locus was relatively resilient to CRISPR/Cas9-based genome editing using single sgRNAs, we identified one potent VLT-specific sgRNA (sgRNA VLT-9) and showed efficient editing of the VZV VLT genomic region when two sgRNAs were combined. This highlights the potential for therapeutic use in humans, where the VLT locus is the only site in the VZV genome that is transcriptionally active during the latent phase, suggesting a relatively open chromatin structure that could allow for SpCas9-sgRNA binding, cleavage and potential disruption of the viral genome. 

In conclusion, this study describes the use of CRISPR/Cas9-based genome editing to generate VZV mutants. Specifically, we applied this method to investigate the role of pVLT and pVLT-ORF63 during lytic VZV infection in vitro. Our data provide valuable insight into the effectiveness of VLT-specific sgRNAs or combinations thereof that can be used to optimize the inactivation of latent VZV genomes in in vitro neuronal latency models and could ultimately be applied in the human host. 

## Figures and Tables

**Table 1:** Overview of viruses recovered from sgRNA VLT-9 expressing cells after plaque purification.

Virus Isolate ^a^	Rescue Cells ^b^	Nucleotide Sequence	Amino Acid Sequence	Mutation Type ^c^	pVLT/pVLT-ORF63 ^d^
VZV EMC-1	n.a.	gatataccaacccttacgaccaatagcaac	PDIPTLTTNSN	n.a.	yes
A-VLT-1	ARPE-19	gatataccaaccctt accaatagcaac	PDIPTLT NSN	Δ1aa	yes
A-VLT-5	ARPE-19	gatataccaaccctt accaatagcaac	PDIPTLT NSN	**Δ1aa**	yes
A-VLT-7	ARPE-19	gatataccaacccttacgaccaatagcaac	PDIPTLTTNSN	**wt**	yes
A-VLT-8	ARPE-19	gatataccaaccctt accaatagcaac	PDIPTLT NSN	Δ1aa	yes
A-VLT-9	ARPE-19	gatataccaaccctt accaatagcaac	PDIPTLT NSN	Δ1aa	yes
P-VLT-3	ARPE-19 pVLT	gatataccaacccttgcggccaatagcaac	PDIPTLADQ-	stop	no
P-VLT-4	ARPE-19 pVLT	gatataccaaccctt accaatagcaac	PDIPTLT NSN	Δ1aa	yes
P-VLT-5	ARPE-19 pVLT	gatataccaacccttac accaatagcaac	PDIPTLPIATLR	**shift**	no
P-VLT-6	ARPE-19 pVLT	gatataccaaccctt accaatagcaac	PDIPTLT NSN	Δ1aa	yes
P-VLT-8	ARPE-19 pVLT	gatataccaaccc aatagcaac	PDIPTQ-	**stop**	no

^a^ Viruses name. ^b^ Cell type used in first plaque purification: parental ARPE-19 or pVLT-expressing ARPE-19. ^c^ Type of mutation: wild-type (no mutation), single amino acid deletion (Δ1aa), insertion of a premature stop codon (stop) and frame-shift mutation (shift). Viruses indicated in green and bold were studied in detail. ^d^ Predicted ability of the virus to encode pVLT/pVLT-ORF63 (variants). n.a.: not applicable.

## Data Availability

All sequencing datasets generated as part of this study are available at the European Nucleotide Archive under the accession: PRJEB47381.

## Supplementary Materials

The following are available online at https://www.mdpi.com/article/10.3390/v13112289/s1, Figure S1: Comparison of infectious foci produced by VZV EMC-1 and VZV pOka derived viruses, Table S1: sgRNA sequences, Table S2: primers used in this study, Table S3: Illumina sequencing of VZV pVLT mutants.
