# Supplementary material for: Mutagenesis of the Varicella-Zoster Virus Genome Demonstrates That VLT and VLT-ORF63 Proteins Are Dispensable for Lytic Infection
Source: Viruses. 2021 Nov 16;13(11):2289. doi: 10.3390/v13112289 (PMC8619377; doi:10.3390/v13112289)
Supplement: Supplementary file 1 [file viruses-13-02289-s001.zip › Supplementary Figure S1.pdf]

SUPPLEMENTARY FIGURE S1

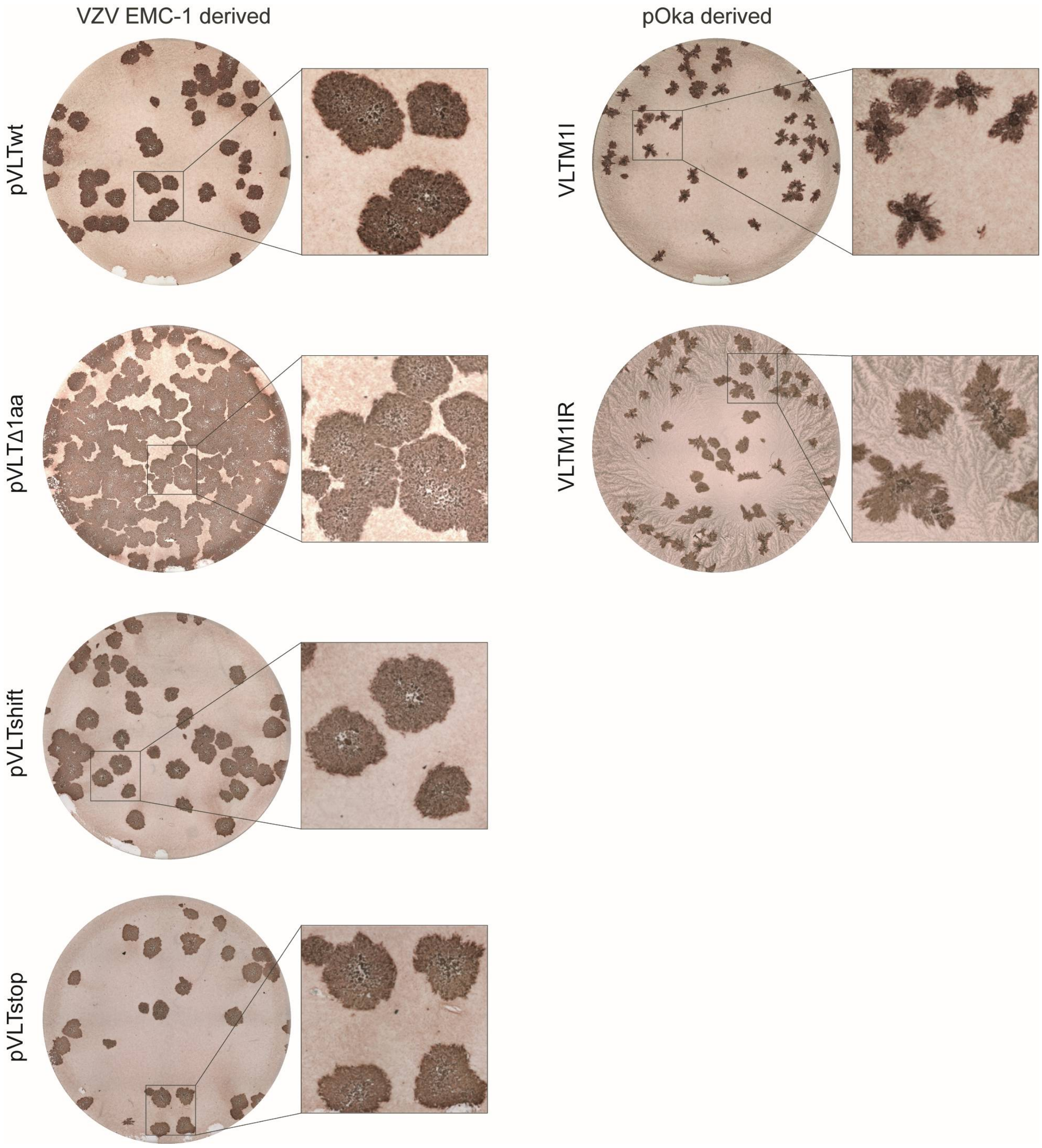

Figure S1: Comparison of infectious foci produced by VZV EMC-1 and VZV pOka derived viruses**.** Infectious focus assay of CRISPR/Cas9-derived (EMC-1 based) viruses (left) and BAC-derived (pOka based) viruses (right) on ARPE-19 cells at six days post-infection. Infected wells were stained for VZV glycoprotein E by immunohistochemistry.
